# Vitamin D Deficiency and Antenatal and Postpartum Depression: A Systematic Review

**DOI:** 10.3390/nu10040478

**Published:** 2018-04-12

**Authors:** Fariba Aghajafari, Nicole Letourneau, Newsha Mahinpey, Nela Cosic, Gerald Giesbrecht

**Affiliations:** 1 Department of Family Medicine and Community Health Sciences, Cumming School of Medicine, University of Calgary, Sunridge Family Medicine Teaching Centre, Calgary, AB T2N 1N4, Canada; 2 Faculty of Nursing and Cumming School of Medicine, University of Calgary, Calgary, AB T2N 1N4, Canada; nicole.letourneau@ucalgary.ca; 3 Life Science Program, Queen’s University, Kingston, ON K7L 3N6, Canada; 16nm5@queensu.ca; 4 Cumming School of Medicine, University of Calgary, Calgary, AB T2N 1N4, Canada; ncosic@ucalgary.ca; 5 Departments of Paediatrics and Community Health Sciences, Cumming School of Medicine, University of Calgary, Calgary, AB T2N 1N4, Canada; ggiesbre@ucalgary.ca

**Keywords:** vitamin D, 25(OH)D, antenatal depression, postnatal depression, depression, pregnancy

## Abstract

Vitamin D has been implicated in antenatal depression (AD) and postpartum depression (PPD) in many studies; however, results have been inconsistent due to the complexity of this association. We searched the MEDLINE, Embase, PsycINFO, and Maternity and Infant Care databases for literature addressing associations between vitamin D and AD and PPD. Two independent authors reviewed titles and abstracts of the search results and selected studies for full review. Data were extracted, and a quality rating was done using the Newcastle–Ottawa Scale (NOS) on the selected studies. A total of 239 studies were identified; 14 were included in the review. The quality assessment of the included studies ranged from moderate to high. Of the studies on PPD, five of nine (55%) showed a significant association between vitamin D and PPD. Five of seven (71%) studies on AD showed a significant association with vitamin D status. As the included studies used different effect estimates and statistical analyses to report the association, it was not possible to transform the existing data into one single effect measure to employ meta-analytic techniques. While results of this systematic review vary, they indicate a significant association between vitamin D status and AD and PD.

## 1. Introduction

Depressive symptoms during and after pregnancy are the leading cause of disease-related disability among women [1,2,3]. Antenatal depression (AD) and postpartum depression (PPD) are common, with a prevalence of 18% and 19%, respectively [4]. Depressive symptoms during and after pregnancy are associated with unfavourable outcomes for mothers and their infants [5]; AD and PPD have been associated with poor cognitive development, behavioural outcomes, and mental and physical health in children [5].

Although biological, psychological, and environmental theories of depression have been advanced, the underlying pathophysiology of depression remains unknown, and it is probable that several different mechanisms are involved [6]. Studies have shown the beneficial effect of dietary factors on depressive symptoms during pregnancy [7,8,9]. Vitamin D is one of those dietary factors and has been suggested to beneficially affect depression in adults [10,11,12]. It has been hypothesized that vitamin D may act as a neuroactive hormone [13,14,15]. Several studies have shown that vitamin D receptors are broadly distributed throughout the human brain [16] and its deficiency alters neuro-transmitters that are known to be involved in depressive symptoms [17]. Most recently, it has been postulated that vitamin D modulates levels of neuronal calcium ions (Ca^2+^) that are responsible for the onset of depressive symptoms. Conversely, a deficiency of vitamin D may lead to an increase in neuronal Ca^2+^, thus increasing depression [13]. In addition, vitamin D may also play a role in neuro-immunomodulation and neuro-plasticity, both of which are proposed mechanisms for the observed effect on mood [17].

Several studies have investigated the association between blood concentration of 25-hydroxyvitamin D (25(OH)D, the vitamin D metabolite that is the best indicator of vitamin D status in the general population [18] and pregnancy mood disorders [19,20,21,22,23,24,25,26,27,28,29,30,31,32]. However, the results of these studies are inconclusive [19,20,21,22,23,24,25,26,27,28,29,30,31,32]. This study aimed to systematically summarize the evidence on the association between 25(OH)D and AD and PPD.

## 2. Materials and Methods

This systematic review was conducted following the Cochrane Collaboration methodology for systematic reviews [33] and the Preferred Reporting Items for Systematic Reviews and Meta-Analyses (PRISMA) reporting guidelines [34].

### 2.1. Search Strategy and Study Selection

In accordance with a protocol developed a priori, we identified all relevant articles regardless of language by searching the MEDLINE, Embase, PsycINFO and Maternity and Infant Care databases from inception to February 2017 in consultation with a senior research librarian. We also scanned the bibliographies of identified articles. To allow a systematic review of all studies assessing the association between serum 25(OH)D concentration and depression during and after pregnancy outcomes, our initial search was not limited to any type of study. The search was broken into three themes:

(1) to identify vitamin D, a Boolean search was performed using the term “or” to explode (search by subject heading) and map (search by keyword) the following MeSH headings: “vitamin D” OR “vitamin D2” OR “vitamin D3” OR “ergocalciferol” OR “cholecalciferol” OR “25-Hydroxyvitamin D” OR “25(OH)D” OR “25(OH)D2” OR “25(OH)D3” OR “3-epi-25 hydroxyvitamin D” or “D2” OR “D3” OR “vitamin D Deficiency” OR “hypovitaminosis D”;
(2) to identify pregnancy-related outcomes, a second Boolean search was performed using the term “or” to explode (search by subject heading) and map (search by keyword) the following MeSH headings: “pregnancy” OR “prenatal” OR “antenatal”;
(3) to identify depression, a third Boolean search was performed using the term “or” to explode (search by subject heading) and map (search by keyword) the following MeSH headings: “depression” OR “postpartum depression” OR “puerperal depression”.

Themes 1, 2, and 3 were combined using the Boolean operator “and” to answer the focus questions (association between vitamin D status and AD and PD). The search results were compiled using citation management software (EndNote version X7; Clarivate Analytics, Philadelphia, PA, USA).

### 2.2. Eligibility Criteria

We determined screening criteria a priori, with the aim of identifying fully published articles that assessed the association of 25(OH)D and AD and PPD. Two reviewers (F.A. and N.L.) screened abstracts and titles to identify articles for further review. Articles were considered for inclusion if they reported on original data from an original study, included outcome of interest (prenatal and postpartum depression), and utilized blood samples during pregnancy that assessed serum 25(OH)D levels. Two independent reviewers (F.A. and N.L.) searched related articles and links. Additional articles were identified by manual search of the references from the key articles selected. Although the search was not limited by language, all articles included in the review were published in English. Two independent reviewers (F.A. and N.L.) independently assessed the abstracts for potential inclusion, fully reviewed the selected articles, and selected the final articles for the systematic review. Studies that were only published as abstracts were excluded. Disagreements were resolved by means of meeting and discussion among all authors to establish a consensus.

### 2.3. Data Extraction and Management

We developed a data extraction form to collect key indicators of each study, including study design, definition of 25(OH)D cut-off levels, 25(OH)D assessment method, gestational age at serum sampling, location and latitude of population, scale and the cut-off used to assess the outcome of interest (AD and PPD), and whether the studies adjusted for potential confounders in their statistical analysis. Articles were categorized on the basis of the outcomes of interest, antenatal depression and PPD. We collected data on definition of these outcomes as reported in the articles. Articles reporting on multiple outcomes were included. Two reviewers (N.M. and N.C.) independently extracted information from each article and compared findings; any discrepancies were resolved by consensus in meetings with all authors. Attempts were made to contact authors of studies with unclear data.

### 2.4. Assessment of Methodological Quality of Included Studies

Two reviewers (F.A. and N.L.) assessed the methodologic quality of included studies using the Newcastle-Ottawa Scale (NOS) for observational studies [35]. This scale rates studies on three major domains: selection, comparability, and the ascertainment of outcome of interest. We identified high-quality choices by answering “Yes” to the questions in each domain. The more Yeses allocated to a study (to a maximum of nine Yeses), the better the quality it was. Although one of the main sources of bias in these studies was confounders, the definition of cases (participants with or without depression) and the outcome assessment (studies using different scales and different cut-off of the scale to measure depression) was also important. At any point, any disagreement between reviewers was resolved by means of meeting and discussion among all authors to establish a consensus.

### 2.5. Data Synthesis

We extracted data from the included studies and prepared the data in table format. The study outcomes are described in the Results section. The included studies used different estimates and statistical analyses to report associations, thus making it impossible to transform the existing data into one single effect measure. As there was huge heterogeneity among the studies, we were unable to employ meta-analytic techniques.

## 3. Results

### 3.1. Description of Included Studies

**Figure 1:**
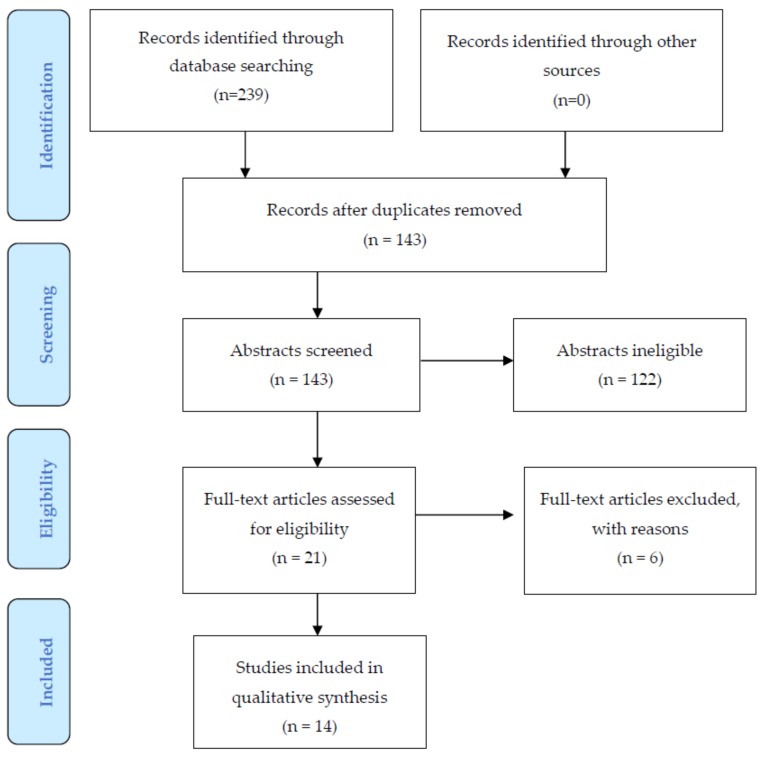
Flowchart for selection of studies.

A total of 239 studies were identified from the combined search and following removal of duplicates 143 were reviewed for titles and abstracts. Ultimately, 21 eligible studies were assessed, and 14 of these were selected for full review, all of which were full-text publications [19,20,21,22,23,24,25,26,27,28,29,30,31,32]. Articles were excluded for the following reasons: a general review [36], study of other nutritional factors, e.g., milk and calcium [37,38], lack of comparison group [39], not measuring the outcome of interest/not population of interest [40,41], or published as an abstract [42] (Figure 1).

In total, 11,888 women were included. Two studies were conducted in southern latitudes between 25 and 32 degrees south [19,25] and the rest were conducted in northern latitudes between 29 and 56 degrees north [20,21,22,23,24,26,27,28,29,30,31,32]. The studies were conducted in Australia [19,25], China [22], Denmark [21], Iran [31], Japan [30], Netherlands [28], Turkey [23], and the United States [20,27,29,32], of which two focused on African-American women [20,29]. Eight of the 14 studies were prospective designs [19,20,22,23,25,26,28,29], of which two were secondary analyses of randomized controlled trials [19,26], four were cross-sectional [24,27,30,32], one was a nested case-control [21], and one was a randomized controlled trial [31].

The predictor measures of 25(OH)D included LC-MS/MS [19,21,27,32], chemiluminescence [20,29,31], radioimmunoassay [24,26], enzyme immunoassay [25,28], E601 modular analyser [22], ELISA [23], and dietary intake [30]. Gestational age at sampling ranged from nine weeks [20,29] to 36 weeks [28], and postnatal sampling occurred between birth [19] and one year postnatal [21]. Five studies employed multiple time points to assess outcome [19,23,24,26,31].

Seven papers focused on only the outcome of PPD [19,20,21,22,23,24,25], five focused on AD [27,28,29,30,32], two focused on AD and PPD [26,31]. A variety of outcome measures were employed. Most utilized the Edinburgh Depression Scale (EPDS) [43,44], employed antenatally [31] and postnatally [19,20,22,23,24,25,26,31], using cut-offs ranging from ≥9 [24], >12 [19] to ≥12 [20,22,23], six questions derived from the EPDS and a cut-off of ≥6 [25], or categorizing outcomes with <9 as low risk, 9–13 as moderate risk, and ≥13 as high risk [31]. Two studies employed the Center for Epidemiological Studies-Depression Scale (CES-D) [45], using cut-offs of ≥16 [29,30]. One employed physician diagnosis or self-report [32], and another employed administrative data reports of women filling prescriptions for anti-depressants within one year of delivery [31]. Other measures included the Beck Depression Inventory (BDI) [46] and Mini International Neuropsychiatric Interview (MINI) [26,47], Depression Anxiety and Stress Scales (DASS-21) [48], and Patient Health Questionnaire Depression Module (PHQ-9) [27,49]. The studies examined a wide range of covariates (e.g., sociodemographics, ethnicity, and BMI), except for one study that relied on randomization to manage potential covariates [31]. See Table 1 and Table 2 for a summary of the studies selected for inclusion.

### 3.2. Methodologic Quality of Included Reviews

When using the NOS for cohort/cross-sectional studies, the quality assessment of the included studies ranged from low scores of 4 [24] and moderate scores of 5–6 [20,25,27,28,30] to high scores of 8–9 [19,22,23,25,29]. For the one case-control study, the NOS score was high [21]. Table 3 shows the NOS quality scores of the included reviews. The quality of the included randomized clinical trial (RCT) [31] was assessed using the Jadad scoring system [50] and was moderate (score of 3 out of 5).

### 3.3. Association between Vitamin D Status and AD

Five of the seven studies (71%) that examined AD as an outcome showed a significant association between lower levels of vitamin D and AD [26,28,29,30,31], while one study showed no association [27]. In contrast, one study found that higher (as opposed to lower) vitamin D concentration was associated with a higher risk of AD at 16 weeks (AOR 1.14, 95% CI 1.02–1.27) [32]. Higher vitamin D was associated with reduced odds of AD at 18.5 weeks (AOR 0.51, 95% CI 0.31–0.72) [30], and 21 weeks gestation (AOR 0.54, 95% CI 0.29–0.99) [29], as well as reduced symptoms of depression at 38–40 weeks gestation (lower EPDS scores, *p* < 0.001) [31]. In one study, vitamin D concentrations <29 nmol/L at 16 weeks of pregnancy was associated with higher odds of AD (AOR 1.48, 95% CI 1.13–1.95) [28]. Of the studies with significant associations, two were considered high quality [28,29]. Significant associations were found with Caucasian [28,32], African American [29,32], Japanese [30], and Iranian [31] samples. As the included studies used different effect estimates and statistical analyses to report associations, it was not possible to transform the existing data into one single effect measure to employ meta-analytic techniques. See Table 1 and Table 2 for details.

### 3.4. Association between Vitamin D Status and PPD

Five of the nine studies (55%) that examined PPD as an outcome showed a significant association between vitamin D in pregnancy and PPD [22,23,24,25,31], while four studies showed no association [19,20,21,26]. Lower vitamin D concentration (<47 nmol/L) was associated with increased odds of PPD at three days postpartum (AOR 2.72, 95% CI 1.42–5.22) [25], and higher levels of vitamin D was associated with decreased odds of PPD three months postpartum (AOR 0.81, 95% CI 0.70–0.92) [22], as well as reduced symptoms at one week, six weeks, and six months postpartum (r = 0.2, *p* = 0.02; r = 0.2, *p* = 0.01; r = 0.3, *p* = 0.01, respectively) [23], four to eight weeks postpartum (*p* < 0.001) [31], and one to seven months postpartum (*p* < 0.02) [24]. One study found an association between lower vitamin D concentration and higher depressive symptom score, but not with diagnosis of major depressive disorders [26]. Of the studies that found significant associations, three were of high quality [22,23,26]. Significant associations were found with Caucasian (American and Australian) [24,25,26], Chinese [22], Iranian [31], and Turkish [23] samples. As the included studies used different effect estimates and statistical analyses to report the association, it was not possible to transform the existing data into one single effect measure to employ meta-analytic techniques. See Table 1 and Table 2 for details.

## 4. Discussion

Available data provide evidence on the potential association between vitamin D status during pregnancy and AD and PPD. We systematically assessed evidence of association between serum 25(OH)D concentration and the risk of AD and PPD, including observational studies and one RCT. Variations in study characteristics, analytical methods, and methodological qualities were identified. To the best of our knowledge, this is the first systemic review examining the association between vitamin D and both AD and PPD. We were not able to employ meta-analytic techniques to this review due to different effect estimates and statistical analyses that these studies used to report the association. This systemic review overall showed a trend toward significant association between vitamin D status during pregnancy and AD and PPD; however, the causality from these findings cannot be inferred due to the observational nature of the studies included in this systematic review.

These findings are of concern, particularly given recent evidence suggesting that 25(OH)D deficiency or insufficiency is common during pregnancy, especially among high-risk groups [51,52,53]. Vitamin D supplementation may be a simple way to reduce the risk of these adverse outcomes. In fact, one RCT [31] showed that supplementation with 2000 IU/day of vitamin D during pregnancy reduces the risk of antenatal depression. This suggests that low levels of 25(OH)D may be a modifiable risk factor in pregnancy, and health care providers should at least be encouraging pregnant women to follow current guidelines on recommended daily allowances for vitamin D [18].

Several mechanisms may explain the observed association between 25(OH)D concentration and risk of depression in pregnancy. Depression is associated with dysregulated hypothalamic-pituitary-adrenal axis function, overactivity of the sympatho-adrenal system, and increased level of inflammatory markers [54]. Vitamin D has been shown to down-regulate inflammatory mediators that have been linked to sickness behaviour, psychosocial stress, and depression [55]. Studies in pregnant women suggest that the effect of low 25(OH)D on PPD symptoms may be potentiated in the presence of inflammation [20]. Vitamin D may also have direct neuroregulatory activity. Vitamin D receptor (VDR) gene polymorphisms in humans have been associated with cognitive impairment and depressive symptoms [56]. In addition, systematic reviews of epidemiological studies have shown that lower concentration of 25(OH)D is correlated with increased risk of depression in adults [10,57].

## 5. Limitations

This systematic review is limited by a lack of or limited adjustment for confounding factors in some of the included studies. Studies have used different assays to measure vitamin D concentrations. A few systematic reviews of vitamin D and disease-related outcomes have found that the method of 25(OH)D measurement was an important determinant of heterogeneity [58,59]. The method of vitamin D measurement is an important factor, as DEQAS (International Vitamin D External Quality Assessment Scheme) has reported a range of inter-method variability for identical blood samples [60]. Currently, the best assay to measure different types of vitamin D including epimers is LC-MS/MS (liquid chromatography-tandem mass spectrometry) [61]. In addition to the inherent flaws in the assay methodology, the studies varied in their definition of cut-offs for 25(OH)D deficiency (>50 nmol/L) and (>75 nmol/L). Moreover, there are other confounding factors that may affect vitamin D status, including exposure to UVB (Ultraviolet B), latitude, season, ethnicity, nutritional status, BMI, and VDR (vitamin D receptor) genotype [62]. Furthermore, there are multiple factors affecting depression during and after pregnancy: age, other comorbidities, education and living situations, social support, and income [4]. These parameters were absent or partially evaluated in most studies included in this systematic review. Finally, in the assessment of antenatal and postnatal depression, discrepancies exist among the studies on the type of assessment tools and the cut-offs they used to describe antenatal or postnatal depression.

## 6. Conclusions

In summary, based on the systemic evaluation of the previous literature in the field, there may be an association between lower vitamin D status and increased risk of depressive symptoms during and after pregnancy. While the quality of the available evidence was not always optimal due to lower methodologic quality of the studies, this review provides an analysis of the methodological issue that future supplementation studies need to consider in their research design.

## Figures and Tables

**Table 1:** Characteristics of included studies.

Author, Year	Location, Latitude	No. of Participants	Age (Years)	Gestational Age at Sampling (Weeks, Unless Otherwise Indicated)	Outcome	Outcome Assessment Scale/Cut-Point
Gould et al., 2015 [19]	Australia, multicenter, 25° S	1040	<24: 25% 25–29: 24% 30–34: 26% >35: 25%	Delivery, cord blood	PPD	EPDS > 12d
Accortt et al., 2016 [20]	Detroit, MI, USA, 42° N	203 African-American	26 ± 6	9.7 ± 3.7	PPD	EPDS ≥ 12
Nielsen et al., 2013 [21]	Denmark, 56° N	Cases: 605 Controls: 875	<25: 14% 26–28: 24% 29–30: 17% 31–32: 24% >34: 22%	Median (IQR) Cases: 24 (24–25) Controls: 25 (24–26)	PPD	Women who filled anti-depressant prescription within 1 year after delivery
Fu et al., 2015 [22]	Beijing, China, 39° N	248 Chinese	Median (IQR) 31 (29–32)	24–48 h after delivery	PPD	EPDS ≥ 12
Gur et al., 2013 [23]	Izmir, Turkey, 38° N	208	28.5	25.2	PPD	EPDS ≥ 12
Murphy et al., 2010 [24]	Charleston, SC, USA, 32° N	97	28.9 ± 5.5	1,2,3,4,5,6,7 m postpartum	PPD	EPDS ≥ 9
Robinson et al., 2014 [25]	Perth, Australia, 31° S	796	<20: 9% 20–29.9: 51% >30: 40%	18	PPD	6 questions derived from EPDS, ≥ 6
Williams et al., 2016 [26]	Michigan, USA, 44° N	126 at risk for depression	30.8 ± 5.04	12–20 34–36	Antenatal depression, PPD	BDI (assess symptoms severity), MINI (diagnose MDD and GAD)
Huang et al., 2014 [27]	Seattle, WA, USA, 47° N	498	33.4 ± 4.2	15.4	Antenatal depression	PHQ-9: Minimal: 0–4 Moderate: 10–14 Moderately severe: 15–19 Severe: 20–27 DASS-21 Depression: Normal: 0–9 Mild: 10–13 Moderate: 14–20 Severe: 21–27 Extremely severe: ≥28
Brandenbarg et al., 2012 [28]	Amsterdam, The Netherlands, 52° N	4389	31 ± 4.8	Median (IQR) 13 (12–14)	Antenatal depression	CES-D ≥ 16
Cassidy-Bushrow et al., 2012 [29]	Detroit, MI, USA, 42° N	203 African-American	26 ± 6	9.5 ± 3.6	Antenatal depression	CES-D ≥ 16
Miyake et al., 2015 [30]	Japan, multicenter, 32° N	1745	31.2 ± 4.3	18.5 ± 5.4 (questionnaire)	Antenatal depression	CES-D (Japanese version) ≥16
Vaziri et al., 2016 [31]	Shiraz, Iran, 29.591° N	153 Vit D: 78 Control: 75	26.31 ± 4.59	26.28, after delivery	Antenatal depression, PPD	EPDS ≥13: severe risk 9–13: moderate risk <9: low risk
Arnold et al., 2013 [32]	Washington, DC, USA, 38° N	MAD: 148 Controls: 554	33.1 ± 5.1	16	Antenatal mood and anxiety disorders	Physician diagnosed or self-reported

**Table 2:** Characteristics of included studies (cont’d).

Author, Year	Time of Outcome Assessment	25(OH)D Quantification Method	25(OH)D Concentration (nmol/L)	Estimates, RR or OR (95% CI)	Adjusted Covariates	Study Design	Results
Gould et al., 2015 [19]	6 w, 6 m postpartum	LC-MS/MS	<25: 8% 25–50: 34% >50: 57%	6 w: ARR: 0.92 (0.84, 1.02) 6 m: ARR: 0.96 (0.88, 1.05)	Age, race, parity, BMI, education, previous depression, smoking, supplement use, season, centre, MSSI	Prospective Secondary analysis of RCT of DHA in pregnancy	No association
Accortt et al., 2016 [20]	4–6 w postpartum	Chemiluminescence immunoassay	32.95 ≥23.46 <50: 85%	β 0.209 *p*: 0.058 No association	Age, marital status, education, smoking, BMI, previous depression Interaction term: vit D status and inflammatory markers	Prospective	No association
Nielsen et al., 2013 [21]	Within 1 year postpartum	LC-MS/MS	Cases: Median (IQR) 55.62 (36.9–74.6) <49: 42% >80: 20% Controls: Median (IQR) 55.60 (37.5–72.4) <49: 42% 50–79: 42% >80: 16%	<50: AOR: 1.13 (0.84–1.51) >80: AOR: 1.53 (1.04–2.26)	Age, season, gestational week at sampling, parity, smoking, SES, BMI, physical activity, social support, multivitamin intake	Nested case-control	No association
Fu et al., 2015 [22]	3 m postpartum	E601 modular analyser (Elecyc)	Median (IQR): 34 (23.5–67.7) <50: 82.6%	AOR: 0.81 (0.70–0.92) *p* < 0.0001	Age, breastfeeding, stressful life event, education, family income, partner support, mode of delivery, planned pregnancy, health problems during pregnancy, marital status, maternal hospital readmission, depression during or before pregnancy	Prospective	Significant association
Gur et al., 2013 [23]	1 w, 6 w, 6 m postpartum	ELISA	55.91 ± 27.95 <25: 11% <50: 40.3% >50: 48.5%	Negative correlation vit D and EPDS: 1 w (r = 0.2, *p* = 0.02) 6 w (r = 0.2, *p* = 0.01) 6 m (r = 0.3, *p* = 0.01)	Age, BMI, season of sampling, supplement use, parity, gestational week at sampling	Prospective	Significant association
Murphy et al., 2010 [24]	1,2,3,4,5,6,7 m postpartum	RIA	≤80: 58% >80: 42%	Adjusted mean EPDS sum scores were lower for group with higher vit D (>80) at all 7 visits (*p* < 0.02)	Age, race, marital status, insurance, season of sampling, supplement use, feeding type, planned pregnancy	Cross-sectional	Significant association
Robinson et al., 2014 [25]	3 d postpartum	Enzyme immunoassay	Q1 <47: 24% Q2 47–58: 23% Q3 59–70: 26% Q4 >70: 26%	4Q: reference 3Q AOR: 1.61 (0.83–3.10) 2Q AOR: 1.37 (0.71–2.63) 1Q AOR: 2.72 (1.42–5.22)	Age, family income, education, season of birth, BMI, SES factors, smoking, alcohol use, HTN of pregnancy, gender of child, NICU admission	Prospective cohort	Significant association
Williams et al., 2016 [26]	12–20 w, 34–36 w, 6–8 w postpartum	RIA (Diasporin)	12–20 w: 70.31 ± 20.59 <50: 16% 34.35 w: 79.47 ± 26.53 <50: 12%	Low vit D at 12–20 w: predictor of BDI at 12–20 w and 34–36 w (*p* < 0.05), but not associated with diagnosis of depression	Serum DHA and EPA level, age, smoking, BMI, anti-depressant medication	Prospective Secondary analysis of RCT	AD: Significant association PPD: No association
Huang et al. 2014 [27]	15.4 w	LC-MS/MS	Mean: 85.85 = 21.71 Q1: 72 Q2: 72.1–85 Q3: 85.1–98 Q4: >98.1	β (95% CI) PHQ-9: 0.019 (−0.020, 0.058) DASS-21: Depression: 0.017 (−0.038, 0.071)	Age, BMI, season, gestational age at sampling, smoking, race, marital status, white race, education	Cross-sectional	No association
Brandenbarg et al., 2012 [28]	Median (IQR) 16 (14–18) w, 16 w	Enzyme immunoassay	57 ± 23.46 <29: 23% 30–50: 21% ≥50: 56%	AOR: vit D deficiency (<29): 1.48 (1.13–1.95) vit D insufficiency (30–50): 1.44 (1.12–1.85)	Age, parity, ethnicity, BMI, smoking, alcohol consumption, education, employment, marital status, wanted pregnancy	Prospective	Significant association
Cassidy-Bushrow et al., 2012 [29]	21 ± 3.8	Chemiluminescence immunoassay	32.95 ± 23.46 <25: 31.5% 25–50: 51%	AOR: 0.54 (0.29–0.99), *p* = 0.046	Age, marital status, education, season of sampling, time between 24(OH)D and CES-D measure	Prospective	Significant association
Miyake et al., 2015 [30]	18.5 ± 5.4	N/A	N/A Dietary intake: 228 ± 120 (IU/day)	4Q (340 IU) AOR: 0.52 (0.30–0.89) 3Q (236 IU) AOR: 0.73 (0.49–1.07) 2Q (184 IU) AOR: 0.79 (0.55–1.11) 1Q (124 IU) AOR: 1:00	Age, gestation, region of residence, number of children, family structure, history of depression, family history of depression, smoking, second-hand smoker, job type, income, education, BMI, intake of SFA, EPA, DHA	Cross-sectional	Significant association
Vaziri et al., 2016 [31]	26–28 w, 38–40 w of gestation, 4–8 w postpartum	Chemiluminescence immunoassay	Baseline: 30.82 ± 17.89 <50: 76.2% 50–75: 19.2% >75: 4.6%	Pairwise comparison: lower EPDS scores at 38–40 and 4–8 w postpartum in vit D group (*p* < 0.001)	N/A	RCT, 2000 IU vit D from 26–28 w to birth vs. placebo	Significant association (AD and PPD)
Arnold et al., 2013 [32]	16 w	LC-MS/MS	MAD: Mean: 78.34 ± 23.46 No MAD: Mean: 72.88 ± 24.96	Higher risk of MAD with higher vit D ≥75 AOR: 1.14 (1.02–1.27) <75 AOR: 0.77 (0.53–1.112)	Age, season, race, BMI Effect modifier: smoking, BMI	Cross-sectional	Significant association

AD: antenatal depression; AOR: adjusted odds ratio; ARR: adjusted relative risk; BDI: Beck Depression Inventory; BMI: body mass index; CES-D: Center for Epidemiologic Studies Depression Scale; CI: confidence internal; DASS-21: Depression, Anxiety and Stress Scale 21-Items; DHA: decosahexaenoic acid; EPA: eicoaspentaenoic acid; EPDS: Edinburgh Depression Scale; ELISA: enzyme-linked immunosorbent assay; GAD: generalized anxiety disorder; HTN: hypertension; IQR: interquartile range; LC-MS/MS: liquid chromatography-tandem mass spectrometry; MAD: mood and anxiety disorders; MDD: major depressive disorder; MINI: Mini International Neuropsychiatric Interview; MSSI: Maternal Social Support Index; N/A: not applicable; NICU: neonatal intensive care unit; OR: odds ratio; PHQ-9: Patient Health Questionnaire Depression Module; PPD: postpartum depression; RCT: randomized clinical trial; RIA: rapid direct radioimmunoassay; RR: relative risk; SES: socioeconomic status; SFA: saturated fatty acid.

**Table 3:** Quality assessment of included studies.

Cohort Studies/Cross-Sectional Studies										
Author, Year	Selection				Comparability		Outcome			
	Representative of Exposed Cohort	Selection of Non-Exposed Cohort	Ascertainment of Exposure	Demonstration That Outcome of Interest Was Not Present at Start of Study	Control For Important Factors	Additional Factors	Assessment of Outcome	Follow-Up	Adequacy of Follow-Up	Score
Gould et al., 2015 [19]	Yes	Yes	Yes	Yes	Yes	Yes	Yes	Yes	Yes	9
Accortt et al., 2016 [20]	No	Yes	Yes	No	Yes	Yes	Yes	Yes	No	6
Fu et al., 2014 [22]	Yes	Yes	Yes	No	Yes	Yes	Yes	Yes	Yes	8
Gur et al., 2013 [23]	Yes	Yes	Yes	Yes	Yes	No	Yes	Yes	Yes	8
Murphy et al., 2010 [24]	No	No	Yes	No	Yes	Yes	Yes	No	No	4
Robinson et al., 2014 [25]	Yes	Yes	Yes	No	Yes	Yes	No, modified	No	Yes	6
Williams et al., 2016 [26]	Yes	Yes	Yes	Yes	Yes	No	Yes	Yes	Yes	8
Huang et al., 2014 [27]	Yes	Yes	Yes	No	Yes	Yes	Yes	No	No	6
Brandenbarg et al., 2012 [28]	Yes	Yes	Yes	No	Yes	Yes	Yes	No	Yes	7
Cassidy-Bushrow et al., 2012 [29]	Yes	Yes	Yes	Yes	Yes	Yes	Yes	Yes	Yes	9
Miyake et al., 2015 [30]	Yes	Yes	Yes	No	Yes	Yes	Yes	No	No	6
Arnold et al., 2013 [31]	Yes	Yes	Yes	No	Yes	Yes	No	No	No	5
**Case Controls**										
**Selection**					**Comparability of Cases and Controls on Basis of Design of Analysis**		**Outcome**			
**Author, Year**	**Adequate Case Definition**	**Representative of Cases**	**Selection of Controls**	**Definition of Controls**			**Ascertainment of Exposure**	**Same Method of Ascertainment for Cases and Controls**	**Non-Response Rate**	**Score**
Nielsen et al., 2013 [21]	Yes	Yes	Yes	Yes	Yes		Yes	Yes	Yes	8
